# Simulation of Organic Liquid Products Deoxygenation by Multistage Countercurrent Absorber/Stripping Using CO_2_ as Solvent with Aspen-HYSYS: Thermodynamic Data Basis and EOS Modeling

**DOI:** 10.3390/molecules26144382

**Published:** 2021-07-20

**Authors:** Elinéia Castro Costa, Welisson de Araújo Silva, Eduardo Gama Ortiz Menezes, Marcilene Paiva da Silva, Vânia Maria Borges Cunha, Andréia de Andrade Mâncio, Marcelo Costa Santos, Sílvio Alex Pereira da Mota, Marilena Emmi Araújo, Nélio Teixeira Machado

**Affiliations:** 1 Graduate Program of Natural Resources Engineering of Amazon, Rua Corrêa N° 1, Campus Profissional-UFPA, Belém 66075-110, Pará, Brazil; elineia.costa.ec@gmail.com (E.C.C.); wasilva89@hotmail.com (W.d.A.S.); dedeiaam@yahoo.com.br (A.d.A.M.); marceloenqui@bol.com.br (M.C.S.); silviomota@unifesspa.edu.br (S.A.P.d.M.); 2 Graduate Program of Chemical Engineering, Rua Corrêa N° 1, Campus Profissional-UFPA, Belém 66075-110, Pará, Brazil; ortizegom@hotmail.com (E.G.O.M.); arci_paiva@hotmail.com (M.P.d.S.); vaniacunha21@hotmail.com (V.M.B.C.); meaaraujo@gmail.com (M.E.A.); 3 Faculty of Sanitary and Environmental Engineering, Rua Corrêa N° 1, Campus Profissional-UFPA, Belém 66075-123, Pará, Brazil

**Keywords:** OLP, thermodynamic data basis, EOS Modeling, process simulation, Aspen-HYSYS

## Abstract

In this work, the thermodynamic data basis and equation of state (EOS) modeling necessary to simulate the fractionation of organic liquid products (OLP), a liquid reaction product obtained by thermal catalytic cracking of palm oil at 450 °C, 1.0 atmosphere, with 10% (wt.) Na_2_CO_3_ as catalyst, in multistage countercurrent absorber/stripping columns using supercritical carbon dioxide (SC-CO_2_) as solvent, with Aspen-HYSYS was systematically investigated. The chemical composition of OLP was used to predict the density (ρ), boiling temperature (T_b_), critical temperature (T_c_), critical pressure (P_c_), critical volume (V_c_), and acentric factor (ω) of all the compounds present in OLP by applying the group contribution methods of Marrero-Gani, Han-Peng, Marrero-Pardillo, Constantinou-Gani, Joback and Reid, and Vetere. The RK-Aspen EOS used as thermodynamic fluid package, applied to correlate the experimental phase equilibrium data of binary systems OLP-_i_/CO_2_ available in the literature. The group contribution methods selected based on the lowest relative average deviation by computing T_b_, T_c_, P_c_, V_c_, and ω. For *n*-alkanes, the method of Marrero-Gani selected for the prediction of T_c_, P_c_ and V_c_, and that of Han-Peng for ω. For alkenes, the method of Marrero-Gani selected for the prediction of T_b_ and T_c_, Marrero-Pardillo for P_c_ and V_c_, and Han-Peng for ω. For unsubstituted cyclic hydrocarbons, the method of Constantinou-Gani selected for the prediction of T_b_, Marrero-Gani for T_c_, Joback for P_c_ and V_c_, and the undirected method of Vetere for ω. For substituted cyclic hydrocarbons, the method of Constantinou-Gani selected for the prediction of T_b_ and P_c_, Marrero-Gani for T_c_ and V_c_, and the undirected method of Vetere for ω. For aromatic hydrocarbon, the method of Joback selected for the prediction of T_b_, Constantinou-Gani for T_c_ and V_c_, Marrero-Gani for P_c_, and the undirected method of Vetere for ω. The regressions show that RK-Aspen EOS was able to describe the experimental phase equilibrium data for all the binary pairs undecane-CO_2_, tetradecane-CO_2_, pentadecane-CO_2_, hexadecane-CO_2_, octadecane-CO_2_, palmitic acid-CO_2_, and oleic acid-CO_2_, showing average absolute deviation for the liquid phase ($AAD_{x}$) between 0.8% and 1.25% and average absolute deviation for the gaseous phase ($AAD_{y}$) between 0.01% to 0.66%.

## 1. Introduction

The OLP obtained by thermal catalytic cracking of lipid-base materials, including vegetable oils [1,2,3,4,5,6,7,8,9,10,11,12,13,14,15,16,17], residual oils [18,19,20,21,22,23,24,25], animal fats [26,27,28], residual animal fat [27], mixtures of carboxylic acids [28,29,30,31,32,33], soaps of carboxylic acids [34,35], and scum, grease & fats [36,37,38], may be used as liquid fuels [1,2,4,5,6,7,8,10,11,13,14,15,16,17,18,19,20,22,26,35,36,37,38,39,40,41], if proper upgrading processes (distillation, adsorption, and liquid-liquid extraction) are applied to remove the oxygenates [6,7,10,16,17,18,19,35,36,37,38,39,40,41].

In order to be used as a liquid fuel, the upgraded and/or de-acidified OLP, must also match the most important physicochemical (acid value, flash point, carbon residue, cloud point, water in sediments, copper corrosiveness), physical (density), and transport properties (kinematic viscosity) to fossil-fuel specifications [10,16,17,18,19,35,36,37,38,39,40,41].

The OLP composed by alkanes, alkenes, ring-containing alkanes, ring-containing alkenes, cycloalkanes, cycloalkenes, and aromatics [4,8,10,16,17,18,19,26,28,29,34,35,36,37,38], as well as oxygenates including carboxylic acids, aldehydes, ketones, fatty alcohols, and esters [4,6,7,8,10,16,17,26,28,34,35,36,37,38,39,40,41]. 

A process with great potential to remove and/or recover oxygenates from OLP (De-acidification of OLP) is multistage gas extraction, using SC-CO_2_ as solvent, based on similar studies reported in the literature [42,43]. However, knowledge of phase equilibrium for the complex system OLP/CO_2_ is necessary.

The knowledge of phase equilibrium data is of fundamental importance for the design of equilibrium-stage separation processes (e.g., multistage gas extraction, absorption, liquid-liquid extraction, distillation), as it provides the thermodynamic basis for the separation process analysis [44]. 

High pressure phase equilibrium data yields information concerning the solubility of the coexisting gas-liquid phases, solvent capacity, compositions of the coexisting phases, distribution coefficients, and selectivity [44]. Those measurements are time consuming, needs special infrastructure (equilibrium cells, sampling units, and compressors), sophisticated chemical analysis (GC-MS, HPLC, etc.), and qualified human resources, thus posing not only a complex experimental task, but also high investment and operational costs [44,45]. 

In this context, the construction of a thermodynamic data basis is necessary for the modeling of complex/multi-component mixtures/CO_2_ using EOS. Process thermodynamic modeling of complex mixtures/CO_2_ using EOS, is a powerful tool to provide preliminary information of high-pressure phase equilibrium of complex multi-component systems/CO_2_, for guiding experimental high-pressure phase equilibrium measurements, as well as to reduce the number of necessary experiments, but not to replace experimental data [45].

Modern design of equilibrium stage processes (e.g., fractionation of multi-component liquid mixtures by multistage countercurrent absorber/stripping columns using supercritical CO_2_ as solvent) requires thermodynamic models capable of predicting the chemical composition of the coexisting phases without the preliminary use of experimental data. In addition, the applied thermodynamic models must be capable to perform accurate computation of mutual solubility’s of the coexisting liquid and gaseous phases in both sub-critical and critical regions [46]. However, simultaneous fulfillment of these requirements is a very difficult and challenging task for EOS [47,48].

Wheat straw was received in chopping lengths of 4–5 cm from the inaccuracy of EOS to predict the chemical composition and the mutual solubility’s of the coexisting phases in both the sub-critical and critical regions for complex systems/CO_2_, may be overcome if phase equilibrium data for some of the binary pairs (multi-component mixture compounds)-_i_/CO_2_ are available in the literature [46].

A key point in this approach is to determine the binary interaction parameters binary $k_{aij}$ and $k_{bij}$ by correlating phase equilibrium data for the binary pairs (multi-component mixture compounds)-_i_/CO_2_ available in the literature [46]. Knowledge of binary interaction parameters makes it possible to construct the matrix of binary interaction parameters [46].

The RK-Aspen EOS with the van der Waals mixing rules and RK-Aspen combining rules for two temperature-independent binary interaction parameters $k_{aij}=k_{aij}^{0}$ and $k_{bij}=k_{bij}^{0}$, and the $k_{ij}$ between two components i and j is a function of the pure component critical properties (T_ci_, T_cj_, P_ci_, P_cj_) and acentric factors (ω_i_, ω_j_). In this sense, it is necessary to compute the critical properties and acentric factors of all the chemical species present in the composition of complex/multi-component mixture [46].

EOS of van der Waals type with van der Waals quadratic mixing rules, including the PR-EOS with van der Waals quadratic mixing rules, PR-EOS with quadratic mixing rules, PR-EOS with a temperature dependent binary interaction parameter $k_{ij}$, computed by a group contribution method, applied to predict high-pressure phase equilibrium for the binary systems carboxylic acids-CO_2_, hydrocarbons-CO_2_, and fat-soluble substances-CO_2_ [45,46,47,48,49,50,51,52,53,54,55]. Most studies analyzed the thermodynamic modeling of binary systems carboxylic acids-CO_2_, alkanes-CO_2_, alkenes-CO_2_, cycloalkanes-CO_2_, cycloalkenes-CO_2_, aromatics-CO_2_, alcohols-CO_2_, fat-soluble substances-CO_2_, and fatty alcohols-CO_2_ [45,46,47,48,49,50,51,52,53,54,55], representing the majority of the binary pairs OLP-_i_/CO_2_, but also complex multi-component systems/CO_2_ [45,46]. In addition, group contribution (GC) method combined with the perturbed-chain SAFT (PC-SAFT) and variable-range SAFT (VR-SAFT) EOS, applied to predict high-pressure phase equilibrium for the binary systems *n*-alkanes/CO_2_ [56]. 

Modeling of high-pressure phase equilibrium includes the application of PR-EOS with van der Waals quadratic mixing rules for the binary systems pentane-CO_2_ and toluene-CO_2_ [49], PR-EOS with van der Waals quadratic mixing rules for the binary systems palmitic acid-CO_2_, oleic acid-CO_2_, linoleic acid-CO_2_, stigmasterol-CO_2_, α-tocopherol-CO_2_, squalene-CO_2_, and the complex multi-component system Soy Oil Deodorizer Distillates (SODD)-SC-CO_2_, lumped as a mixture of key compounds palmitic acid, oleic acid, linoleic acid, stigmasterol, α-tocopherol, and squalene [45], PR-EOS with quadratic mixing rules for the binary systems toluene-CO_2_, benzene-CO_2_, and *n*-hexane-CO_2_ [50], PR-EOS with a group contribution method to estimate the binary interaction parameters k_ij_ for 54 (fifty four) binary systems hydrocarbons-CO_2_ [47], PPR78 EOS with temperature dependent k_ij_ calculated using group contribution method to systems containing aromatic compounds-CO_2_ [48], cubic EOS (PR, 3P1T, and PR-DVT), and EOS with association term (PR-CPA EOS, AEOS, SAFT, and SAFT-CB) for the binary systems *o*-cresol-CO_2_, *p*-cresol-CO_2_, and ternary system *o*-cresol-*p*-cresol-CO_2_ [51], SRK EOS with association model to correlate the solubility’s of fatty acids (myristic acid, palmitic acid and stearic acid) and fatty alcohols (1-hexadecanol, 1-octadecanol, and 1-eicosanol in SC-CO2 [52], RK-Aspen and PR-BM EOS for the binary systems oleic acid-CO_2_, triolein-CO_2_, using the ASPEN-plus^®^ software to predict high-pressure phase equilibrium of multicomponent mixture vegetable oil-CO_2_, lumped as a mixture of key compounds oleic acid and triolein, thus represented by the ternary system oleic acid-triolein-CO_2_ [53], RK-Aspen, PR-BM and SR-POLAR EOS for the binary systems *n*-dodecane-CO_2_, 1-decanol-CO_2_, and 3,7-dimethyl-1-octanol-CO_2_, for the ternary system *n*-dodecane-1-decanol-CO_2_, *n*-dodecane-3,7-dimethyl-1-octanol-CO_2_, 3,7-dimethyl-1-octanol-1-decanol-CO_2_, and the quaternary system *n*-dodecane-3,7-dimethyl-1-octanol-1-decanol-CO_2_, using the ASPEN-plus^®^ software to predict high-pressure phase equilibrium of multi-component mixture *n*-dodecane-3,7-dimethyl-1-octanol-1-decanol-CO_2_ [54], PPC-SAFT EOS to predict the global behavior of the binary pair *n*-alkanes-CO_2_ [55], GC-SAFT EOS (Perturbed-Chain SAFT and Variable-Range SAFT) for the binary systems *n*-alkanes-CO_2_, including propane-CO_2_, *n*-butane-CO_2_, *n*-pentane-CO_2_, *n*-hexane-CO_2_, *n*-heptane-CO_2_, *n*-octane-CO_2_, *n*-decane-CO_2_, *n*-dodecane-CO_2_, *n*-tetradecane-CO_2_, *n*-eicosane-CO_2_, *n*-docosane/CO_2_, *n*-octacosane/CO_2_, *n*-dotriacontane/CO_2_, *n*-hexatriacontane/CO_2_, and *n*-tetratriacontane-CO_2_ [56], RK-Aspen for the binary systems organic liquid products compounds-i-CO2, including undecane-CO_2_, tetradecane-CO_2_, pentadecane-CO_2_, hexadecane-CO_2_, octadecane-CO_2_, palmitic acid-CO_2_, and oleic acid-CO_2,_ using the Aspen-HYSYS software to predict high-pressure phase equilibrium of multi-component system PLO-SC-CO_2_ [46].

In this work, the thermodynamic data basis and EOS modeling necessary to simulate the fractionation of OLP in multistage countercurrent absorber/stripping columns using SC-CO2 as solvent, with Aspen-HYSYS was systematically constructed. The physical (ρ), critical properties (T_b_, T_c_, P_c_, V_c_), and acentric factor (ω) of all the compounds present in OLP predicted by the group contribution methods of Marrero-Gani, Han-Peng, Marrero-Pardillo, Constantinou-Gani, Joback and Reid, and Vetere. The RK-Aspen applied to correlate the experimental phase equilibrium data of binary systems organic liquid products compounds (OLP)-_i_-CO_2_ available in the literature. The regressions show that RK-Aspen EOS was able to describe the experimental phase equilibrium data for all the binary pairs (multi-component mixture compounds)-_i_-CO_2_ under investigation.

## 2. Modeling and Simulation Methodology

### 2.1. Thermodynamic Modeling

#### 2.1.1. Prediction of Thermo-Physical (T_b_), Critical Properties (T_c_, P_c_, V_c_), and Acentric Factor (ω) of OLP Compounds

Predictive methods selected by considering their applicability to describe the chemical structure of molecules, including the effects of carboxylic acids and hydrocarbons chain length and molecular weight, and simplicity of use.

Experimental data of normal boiling temperature (T_b_) and critical properties (T_c_, P_c_, V_c_) of carboxylic acids [57,58], esters of carboxylic acids [57,58], hydrocarbons [59,60], and alcohols [58,59,60,61], reported by Ambrose and Ghiasse [57], Simmrock et al. [58], Danner and Daubert [59], Yaws [60], and Teja et al. [61], as well as vapor pressure (P^Sat^) data reported by Ambrose and Ghiasse [57], and Boublik et al. [62], used to evaluate all the methods applied to predict the thermo-physical (T_b_), critical properties (T_c_, P_c_, V_c_), and acentric factor (ω) of OLP compounds described in Table S1 (Supplementary Material).

Based on the chemical composition of OLP described in Table S1, experimental data for critical properties available in the literature selected to the following class of hydrocarbons including alkanes from C_2_-C_20_, cyclic from C_3_-C_17_, alkenes with only one double bound from C_4_-C_20_, and aromatics from C_6_-C_15_, carboxylic acids of linear chain length from C_1_-C_10_, as well as C_16_, C_18_, C_20_, and C_22_, carboxylic acids with one or two double bounds including C_16:1_, C_16:2_, C_18:1_, C_18:2_, C_20:1_, C_20:2_, C_22:1_, C_22:2_, alcohols of linear chain length from C_2_-C_10_.

##### Methods to Predict Thermo-Physical (T_b_) and Critical Properties (T_c_, P_c_, V_c_)

The predictive methods by Joback and Reid [63], Constantinou-Gani [64], Marrero-Marejón and Pardillo-Fontdevila [65], and Marrero-Gani [66] applied to estimate the normal boiling temperature (T_b_) and critical properties (T_c_, P_c_, V_c_) of all the compounds present in OLP. Table 1 presents the equations of all the predictive methods applied to compute T_b_, T_c_, P_c_, and V_c_ [63,64,65,66].

The method by Constantinou-Gani [64], is based only on the molecular structure of molecules, being applied in two levels: the first level treats simple functional groups, also called first order groups, and the second level treats the second order groups, formed by blocks of first order groups. In the equations described in Table 1, T_b1i_, T_c1i_, P_c1i_ and V_c1i_, represent the group contribution of first order level for the corresponding properties, and N_i_ how many times the group i occurs in the molecule. In a similar way, T_b2j_, T_c2j_, P_c2j_ and V_c2j_ represents the group contributions of second order level, and M_j_ how many times the group j occurs in the molecule. 

Marrero-Gani [66], proposed a method analogous to that of Constantinou-Gani [64], in which a group contribution of third order is added, whereas T_b3k_, T_c3k_, P_c3k_ and V_c3k_ represent these contributions, and O_k_ how many times the group k occurs in the molecule.

Joback and Reid [63], proposed a method to estimate the normal boiling temperature (T_b_) and critical properties (T_c_, P_c_, V_c_) using group contribution, where Ʃ symbolizes the sum of all the contributions of each group corresponding to the parts of a molecule. To compute the critical temperature (T_c_), Joback and Reid [63] proposed a method dependent on the normal boiling temperature (T_b_). 

By the method of Joback and Reid [63], n_i_ is the number of contributions, while T_bi_ and T_ci_ are the normal boiling temperature and critical temperature associated to the i-th group contribution. To compute the critical pressure (P_c_), the method by Joback and Reid [63], considers the number of atoms within the molecule, where n_A_ specifies the number of atoms in the molecule, and P_ci_ the critical pressure associated to the i-th group contribution.

Marrero-Pardillo [65], proposed a method to predict the normal boiling temperature (T_b_) and critical properties (T_c_, P_c_, V_c_) of pure organic molecules that uses a novel structural approach. This methodology uses the interactions between the groups of charges within the molecule, instead of the simple group contribution. To estimate the critical pressure (P_c_), and likewise the method by Joback and Reid [63], this method also considers the number of atoms in the molecule.

##### Methods Selected to Predict the Acentric Factor (ω)

The prediction of acentric factor performed by using direct group contribution methods as described by Costantinou et al. [67] and Han and Peng [68], as well as an indirect method using its definition from vapor pressure data, based on the proposal of Araújo and Meireles [69]. In this case, the correlation by Vetere [70] was used, making it possible to estimate the vapor pressure from molecular structure. Experimental values for acentric factors obtained as the follows:

I. Predicted by using experimental data of critical properties, and experimental data of vapor pressure at T_r_ = 0.7 [71];
II. Predicted by using experimental values of critical properties and vapor pressure data at T_r_ = 0.7, computed with Wagner’s equation [72], and the parameters obtained from experimental data fitting.

#### 2.1.2. Statistical Analysis of Predicted Thermo-Physical Property (T_b_), Critical Properties (T_c_, P_c_, V_c_), and Acentric Factor (ω) of OLP Compounds

The same criteria used by Melo et al. [73] and Araújo and Meireles [69] were used to select the best methods to predict the thermo-physical property, critical properties, and acentric factor of OLP compounds. The criteria based on statistical analysis (measurements of central tendency and dispersion).

The decisive criteria to select the best prediction methods for the thermo-physical properties, critical properties, and acentric factor are the measurement of central tendency, represented by the average relative deviation (ARD), the dispersion of deviations (R), and the standard deviation (S), using the procedures as follows:

1. The lower values for the average relative deviation (ARD) and standard deviation (S) define the best methods;
2. In cases where the lower average deviation corresponds to the higher standard deviation, or vice versa, the method is selected by the lower range of deviation (R).

The predicted data for the thermo-physical property, critical properties, and acentric factor computed by the methods and procedures described in Methods to Predict Thermo-Physical (T_b_) and Critical Properties (T_c_, P_c_, V_c_) and Methods Selected to Predict the Acentric Factor (ω), analyzed on basis its consistency related the physicochemical behavior expected for homologous series. 

This test applied to hydrocarbons [61], by relating the thermo-physical property, critical properties, and the acentric factor with the number of carbons in carbon chain length or molecular weight of hydrocarbons.

#### 2.1.3. Correlation of Phase Equilibrium Data for the Binary System OLP Compounds-_i_-CO_2_

##### EOS Modeling

The thermodynamic modeling applied to describe the OLP fractionation in a multistage countercurrent absorber/stripping column using SC-CO_2_ as solvent, performed by the Redlich-Kwong Aspen EOS. 

The RK-Aspen EOS equation of state applied to correlate the binary systems organic liquid products compounds-_i_-CO_2_ available in the literature, as described in Table 2. The RK-Aspen EOS with the van der Waals mixing rules and RK-Aspen combining rules for two temperature-independent binary interaction parameters, described in details by Table 2.

Where $k_{aij}=k_{aij}^{0}$ and $k_{bij}=k_{bij}^{0}$ are the binary interaction parameters, considered as temperature-independent. The RK-Aspen binary interaction parameters obtained using the Aspen Properties computational package from Aspen Plus. The program uses the Britt-Lueck algorithm, with the Deming parameters initialization method, to perform a maximum like-hood estimation of the following objective function, described by Equation (1).

(1) $$OF=\underset{i}{\sum }{\left(\frac{T^{e}-T^{c}}{\sigma_{T}}\right)}^{2}+\underset{i}{\sum }{\left(\frac{P^{e}-P^{c}}{\sigma_{P}}\right)}^{2}+\underset{i}{\sum }{\left(\frac{x_{i}^{e}-x_{i}^{c}}{\sigma_{x}}\right)}^{2}+\underset{i}{\sum }{\left(\frac{y_{i}^{e}-y_{i}^{c}}{\sigma_{y}}\right)}^{2}$$

where, $x_{i}^{e}$ and $y_{i}^{e}$ are the experimental compositions of i-th compound in the coexisting liquid and gaseous phases, respectively, and σ the standard deviations, applied to the state conditions (T, P) and $x_{i}^{c}$ and $y_{i}^{c}$ compositions of i-th compound predicted with EOS. The average absolute deviation (AAD) computed to evaluate the agreement between measured experimental data and the calculated/predicted results for all the binary systems investigated.

##### High-Pressure Equilibrium Data for the Binary Systems OLP Compound-_i_-CO_2_

Table 3 shows the experimental high-pressure gaseous-liquid equilibrium data for the binary systems OLP compound-_i_-CO_2_ used to compute the binary interaction parameters. For the binary pairs OLP compounds-_i_-CO_2_ not available in the literature, $k_{aij}=k_{aij}^{0}$ and $k_{bij}=k_{bij}^{0}$ were set equal to zero in the matrix of binary interaction parameters.

Because high-pressure phase equilibrium data for the complex system OLP-CO_2_ is not available in the literature, the proposed methodology was tested to simulate the thermodynamic modeling by de-acidification of olive oil, represented by a quaternary model mixture oleic acid-squalene-triolein-CO_2_. 

Table 4 summarizes the experimental high-pressure gaseous-liquid equilibrium data for the binary systems olive oil key (oleic acid, squalene, triolein) compounds-_i_-CO_2_ used to compute the binary interaction parameters.

##### Schematic Diagram of Phase Equilibria Data Correlation

**Figure 1:**
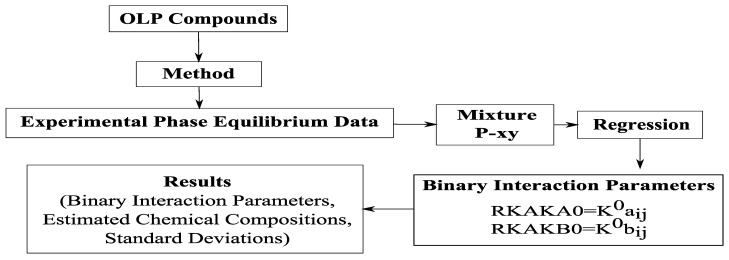
Simplified schematic diagram of the main correlation steps of phase equilibrium data for the binary system organic liquid products compounds-_i_-CO_2_, and the binary system olive oil key (oleic acid, squalene, triolein) compounds-_i_-CO_2_, performed by using the Aspen Properties^®^.

The Aspen Properties^®^ package program used for the regression of experimental phase equilibrium data described in Table 3 and Table 4. Figure 1 illustrates the simplified schematic diagram of the main correlation steps of phase equilibria data for the binary system OLP compounds-_i_-CO_2_, and the binary system olive oil key (oleic acid, squalene, triolein) compounds-_i_-CO_2_, performed by using the Aspen Properties^®^.

The program provides several options showing how to perform regression, including several different types of objective functions. The default objective function is the Maximum likelihood objective function, given by Equation (1). To obtain the binary interaction parameters in Aspen Properties^®^, the following procedure was applied, regardless the type of system and model to which data will be correlated.

1. Choice of components;
2. Specification of the method (where the model applied for the regression of the experimental data is chosen);
3. Introduction or choice of experimental data (T-xy, P-xy, TP-x, T-x, TP-xy, T-xx, P-xx, TP-xx, TP-xxy, etc.) depending on the type and information of the system; at this stage it is possible to either search for the compounds from the Aspen Properties^®^ data base or enter experimental data manually;
4. Regression of data: In this step the type of parameter, the parameters (according to the coding of the program) to be adjusted/correlated, the initial estimate and the limits for the regression chosen.

## 3. Results and Discussions

### 3.1. Prediction of Thermo-Physical Properties and the Acentric Factor of OLP Compounds

#### 3.1.1. Normal Boiling Temperature (T_b_) of OLP Compounds

The most indicated methods, consistent with the selection criteria described in Section 2.1.2, adopted to estimate the normal boiling temperature (T_b_) of hydrocarbons classes present in OLP, illustrated in Table 5. For the *n*-alkanes and alkenes, the method by Marrero-Gani [66], provided the best correlation/regression to experimental data, while the method by Constantinou-Gani [64], shows the best correlation/regression for unsubstituted and substituted cyclics, and that by Joback and Reid [63], was the best for aromatics. Kontogeorgis and Tassios [86], reported that Joback and Reid [63] method was not suitable to estimate critical properties of alkanes of high molecular weight and selected Constantinou-Gani [64], as the best method.

#### 3.1.2. Critical Temperature (T_c_) of OLP Compounds

Table 6 shows the selected methods to predict the critical temperature (T_c_) of hydrocarbons classes (*n*-alkanes, alkenes, unsubstituted cyclics, substituted cyclics, and aromatics), present in OLP. The method by Marrero-Gani [66], is the most suitable for *n*-alkanes, alkenes, unsubstituted and unsubstituted cyclic hydrocarbons, as it showed the best correlation/regression to experimental data, while that by Constantinou-Gani [64], provided the best correlation/regression to experimental data for aromatics. Owczarek and Blazej [87] applied the methods by Joback and Reid [63] and Constantinou-Gani [64], to predict the critical temperature (T_c_) of substituted and unsubstituted cyclic hydrocarbons, reporting deviations of 0.93% and 0.82%, respectively, when using the method by Joback and Reid [63], as well as deviations of 1.77% and 2.00%, respectively, with the method by Constantinou-Gani [64]. The results showed that computed deviations of substituted and unsubstituted cyclic hydrocarbons were 1.41% and 1.69%, respectively, when using the method by Joback and Reid [63], as well as 0.79% and 3.08%, with the method by Constantinou-Gani [64], higher than that described in Table 6, when using the method by Marrero-Gani [66]. The method by Constantinou-Gani [64] is the most suitable for aromatics. As by the estimation of normal boiling temperature (T_b_), prediction of critical temperature (T_c_) of aromatic hydrocarbons included also *n*-alkyl-benzenes, alkyl-benzenes, poly-phenyls, as well as condensed polycyclic aromatics.

#### 3.1.3. Critical Pressure (P_c_) of OLP Compounds

The most indicated methods to estimate the critical pressure (P_c_) of hydrocarbons functions present in OLP are illustrated in Table 7. For *n*-alkanes, the method by Marrero-Padillo [65], provided the best results, while that by Marrero-Gani [66], selected for alkenes. For unsubstituted cyclic, the method by Joback and Reid [63], was selected. For substituted cyclic, the method by Constantinou-Gani [64], was selected. By predicting the critical pressure (P_c_) of aromatics, only the alkyl-benzenes were considered, being the method by Marrero-Gani [66], the best one. This is due to the high average relative deviation obtained for polycyclic condensates and poly-phenyls using all the methods described in Table 1, with ADR higher than 15%, reaching for some cases (m-terphenyl-Cas 92-06-8) 45%. In this sense, none of the methods evaluated showed good precision to estimate the critical pressure (P_c_) of polycyclic condensates and poly-phenyls aromatic.

#### 3.1.4. Critical Volume (V_c_) of OLP Compounds

Table 8 shows the selected methods to predict the critical volume (V_c_) of hydrocarbons classes (*n*-alkanes, alkenes, unsubstituted cyclics, substituted cyclics, and aromatics), present in OLP. The method by Marrero-Gani [66], is the most suitable for *n*-alkanes and substituted cyclic hydrocarbons, as it showed the best correlation/regression to experimental data, while that Joback and Reid [63], selected for unsubstituted cyclic hydrocarbons. The method by Marrero-Pardillo [65], selected for alkenes, while that by Constantinou-Gani [64], provided the best correlation/regression to experimental data for aromatics.

#### 3.1.5. Acentric Factor (ω) of OLP Compounds

The selected methods to estimate the acentric factor (ω) of hydrocarbons classes present in OLP illustrated in Table 9. For *n*-alkanes and alkenes, the method by Han-Peng [68], provided the best results, while the indirect method by Vetere [70], was selected for unsubstituted cyclic, unsubstituted cyclic and aromatics.

### 3.2. Thermodynamic Modeling of Phase Equilibrium Data for the Binary System OLP Compounds-_i_/CO_2_

#### 3.2.1. Estimation of Thermo-Physical (T_b_), Critical Properties (T_c_, P_c_, V_c_), and Acentric factor (ω) of OLP Compounds

Table S2 (Supplementary Material) shows the estimated values of thermo-physical (T_b_), critical properties (T_c_, P_c_, V_c_), and acentric factor (ω) of OLP compounds, recommended for the main chemical compounds present in the OLP obtained by thermal-catalytic cracking of palm oil, as described by Mâncio et al. [17]. The prediction of the normal boiling temperature and critical properties of carboxylic acids and esters of carboxylic acids followed the recommendations of Araújo and Meireles [69], and for estimation of acentric factor (ω), the indirect method proposed by Ceriani et al. [88]. This method makes use of group contributions with high similarities to the molecular structure of carboxylic acids and esters of carboxylic acids. In addition, the method proposed by Ceriani et al. [88], also applied for estimation of critical properties of ketones, while the method of Nikitin et al. [89], applied for alcohols.

#### 3.2.2. Thermo-Physical (T_b_), Critical Properties (T_c_, P_c_, V_c_), and Acentric Factor (ω) of Olive Oil Key (Oleic Acid, Squalene, Triolein) Compounds

The estimated values of thermo-physical (T_b_), critical properties (T_c_, P_c_, V_c_), and acentric factor (ω) of olive oil key (oleic acid, squalene, triolein) compounds summarized in Table 10. The values for the thermo-physical (T_b_), critical properties (T_c_, P_c_, V_c_), and acentric factor (ω) of olive oil model mixture compounds (oleic acid, squalene, triolein) are those predicted by the authors described in Table 4.

#### 3.2.3. Estimation of RK-Aspen EOS Temperature-Independent Binary Interaction Parameters for the Binary Systems Hydrocarbons-_i_-CO_2_ and Carboxylic Acids-_i_-CO_2_

Table 11 presents the RK-Aspen EOS temperature-independent binary interaction parameters adjusted with experimental phase equilibrium data for the binary systems hydrocarbons-_i_-CO_2_ and carboxylic acids-_i_-CO_2_, as well as the absolute mean deviation (AAD) between experimental and predicted compositions for both coexisting liquid and gaseous phases. The regressions show that RK-Aspen EOS was able to describe the high-pressure gaseous-liquid phase equilibrium data for all the systems investigated.

#### 3.2.4. Estimation of RK-Aspen EOS Temperature-Independent Binary Interaction Parameters for the Binary Systems Olive Oil Key (Oleic Acid, Squalene, Triolein) Compounds-_i_-CO_2_

Table 12 summarizes the RK-Aspen EOS temperature independent binary interaction parameters adjusted to experimental high-pressure phase equilibria of olive oil key (oleic acid, squalene, triolein) compounds-_i_-CO_2_, used as test system to simulate the thermodynamic modeling by de-acidification of olive oil, represented by a quaternary model mixture oleic acid-squalene-triolein-CO_2_. 

##### Equation of State (EOS) Modeling for the Binary Systems Olive Oil Key (Oleic Acid, Squalene, Triolein) Compounds-_i_-CO_2_

**Figure 2:**
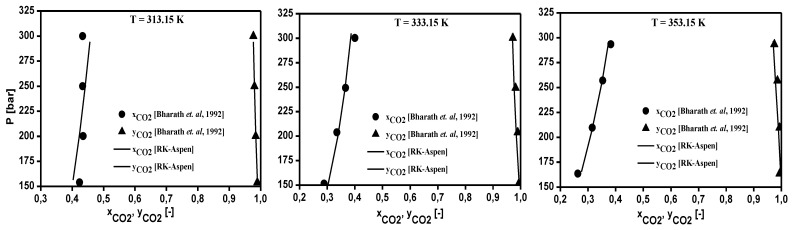
Experimental and predicted high-pressure phase equilibrium for the system oleic acid-CO_2_ (Bharath et al., 1992).

**Figure 3:**
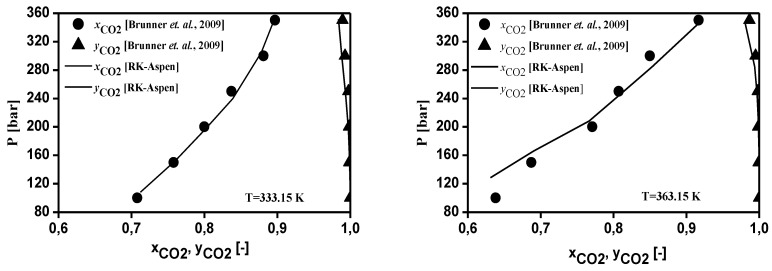
Experimental and predicted high-pressure phase equilibrium for the system squalene-CO_2_ (Brunner et al., 2009).

**Figure 4:**
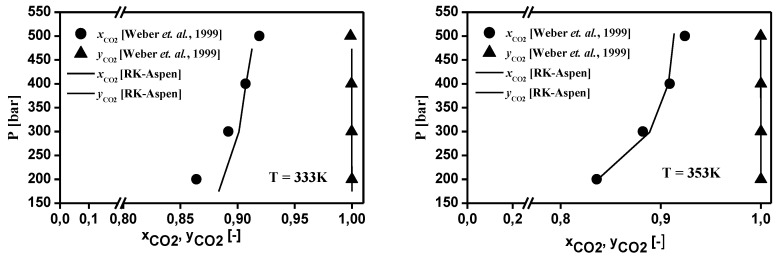
Experimental and predicted high-pressure phase equilibrium for the system triolein-CO_2_ (Weber et al., 1999).

The thermodynamic modeling for the binary systems olive oil key (oleic acid, squalene, triolein) compounds-_i_-CO_2_ performed with RK-Aspen EOS with the van der Waals mixing rules and RK-Aspen combining rules for two temperature-independent binary interaction parameters. The EOS modeling described in form P-x_CO2_,y_CO2_ diagram showing a comparison between predicted and experimental high-pressure equilibrium data for the binary systems oleic acid-CO_2_ (Bharath et al., 1992), squalene-CO_2_ (Brunner et al., 2009), and triolein-CO_2_ (Weber et al., 1999), as shown in Figure 2, Figure 3 and Figure 4, respectively. The regressions show that RK-Aspen EOS was able to describe the high pressure equilibrium data for the binary systems olive oil key (oleic acid, squalene, triolein) compounds-_i_-CO_2_.

##### Simulation Modeling for the Model System Olive Oil Key (Oleic Acid-Squalene-Triolen-CO_2_

Because high-pressure phase equilibrium data for the complex system OLP-CO_2_ is not available in the literature, the proposed methodology tested to simulate the thermodynamic modeling by de-acidification of olive oil, represented by a quaternary model mixture oleic acid-squalene-triolein-CO_2_. 

Table 13 presents the RK-Aspen EOS temperature independent binary interaction parameters adjusted to the experimental high-pressure equilibrium data for the multicomponent olive oil-CO_2_, described in Table 14 [90], and represented in this work as a multicomponent model mixture oleic acid-squalene-triolein-CO_2_. In addition, Table 13 presents the root-mean-square deviation (RMSD) between the multicomponent experimental high-pressure equilibrium data and the computed results for the coexisting gaseous-liquid phases.

The State conditions (T, P) by the experimental high-pressure equilibrium data for the multicomponent olive oil-CO_2_, described in Table 14.

Table 15 presents the average absolute deviation (AAD) between the predicted and experimental high-pressure phase equilibrium data for the model systems oleic acid(1)-squalene(3)-triolein(2)-CO_2_(4).

Table 16 presents the RK-Aspen EOS temperature independent binary interaction parameters adjusted in this work to experimental high-pressure equilibrium for the system olive oil-CO_2_ at 313 K with 2.9 and 7.6 [wt.%] FFA. The RK-Aspen EOS was able to describe the high-pressure phase equilibria of multicomponent system olive oil-CO_2_ [90], showing RMSD between 3E-07 to 0.0138 for the liquid phase and between 0.0009 to 2E-04 for the gaseous phase, by considering the system was represented by the multicomponent model mixture triolein-squalene-oleic acid-CO_2_.

**Figure 5:**
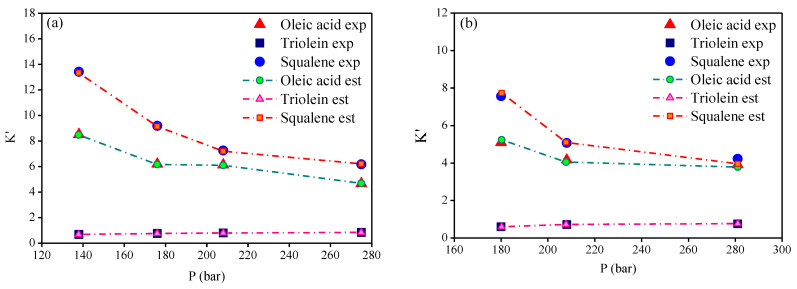
Experimental and estimated distribution coefficients K_i_, expressed in CO_2_-free basis, at 313 K with (**a**) 2.9 and (**b**) 7.6 [wt.%] of FFA.

**Figure 6:**
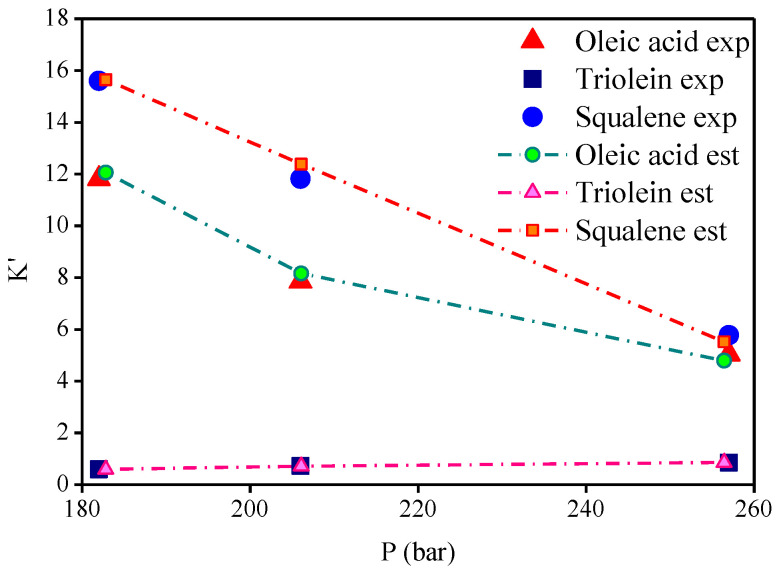
Experimental and estimated distribution coefficients K_i_, expressed in CO_2_-free basis, at 323 K with 2.9 [wt.%] of FFA.

**Figure 7:**
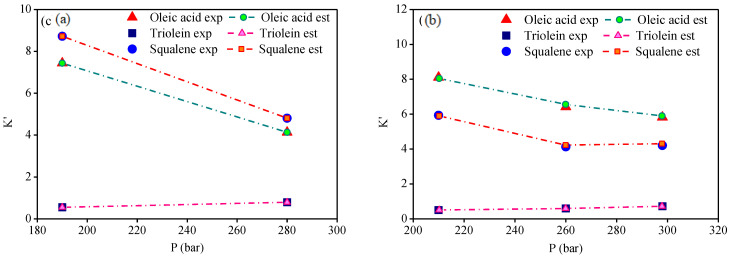
Experimental and estimated distribution coefficients K_i_, expressed in CO_2_-free basis, at (**a**) 338 and (**b**) 353 K with 5.2 [wt.%] of FFA.

The distribution coefficients-*Ki* of key compounds by the experimental high-pressure phase equilibria for the multicomponent system olive oil-CO_2_, described on solvent free basis, as shown in Table 17. Table 17 presents the experimental distribution coefficients of FFA (l), triglyceride (2), and squalene (3) and the estimated distribution coefficients computed using the binary interaction parameter presented in Table 13. The results show the precision of RK-Aspen EOS to describe the multicomponent system for the state conditions (T, P), and free fatty acid (FFA) content in feed. The distribution coefficients described on a solvent free basis provide information about the phase in which the compounds are preferably enriched in the extract (Ki > 1) or in the bottoms (Ki < 1). Figure 5, Figure 6 and Figure 7 show the distribution coefficients for the key compounds of olive oil computed on CO_2_ free basis. The results show that FFA and squalene are preferably enriched in the extract (Ki > 1), while the triolein is enriched in the bottoms (Ki < 1) by de-acidification of olive oil using SC-CO_2_ in countercurrent packed columns.

## 4. Conclusions

The EOS modeling described in form P-x_CO2_,y_CO2_ diagram for the binary systems oleic acid-CO_2_ (Bharath et al., 1992), squalene-CO_2_ (Brunner et al., 2009), and triolein-CO_2_ (Weber et al., 1999), shows that RK-Aspen EOS was able to describe the high pressure equilibrium data for the binary systems olive oil key (oleic acid, squalene, triolein) compounds-_i_-CO_2_.

The RK-Aspen EOS was able to describe the high-pressure phase equilibria of multicomponent system olive oil-CO_2_ [90], showing RMSD between 3 × 10^−7^ to 0.0138 for the liquid phase and between 0.0009 to 2 × 10^−4^ for the gaseous phase, by considering the system was represented by the multicomponent model mixture triolein-squalene-oleic acid-CO_2_.

The proposed methodology proved to simulate with high accuracy the thermodynamic modeling by de-acidification of olive oil, represented by a quaternary model mixture oleic acid-squalene-triolein-CO_2_, and hence can be applied to simulate the fractionation of OLP in multistage countercurrent absorber/stripping columns using SC-CO_2_ as solvent, with Aspen-HYSYS.

## Figures and Tables

**Table 1:** The equations used to predict/estimate the thermo-physical (T_b_) and critical properties (T_c_, P_c_, V_c_) of all the compounds present in OLP, by the methods of Joback and Reid [63], Constantinou and Gani [64], Marrero-Marejón and Pardillo-Fontdevila [65] and Marrero and Gani [66].

Constantinou-Gani [64]	Marrero-Gani [66]
$T_{b}=204.359\;ln\left(\sum_{i}N_{i}\left(T_{b1i}\right)+W\sum_{j}M_{j}\left(T_{b2j}\right)\right)$	$T_{b}=222.543\;ln\left(\sum_{i}N_{i}T_{b1i}+\sum_{j}M_{j}T_{b2j}+\sum_{k}O_{k}T_{b3k}\right)$
$T_{c}=181.128\;\ln\left(\sum_{i}N_{i}T_{c1i}+W\sum_{j}M_{j}T_{c2j}\right)$	$T_{c}=231.239\ln\left(\sum_{i}N_{i}T_{c1i}+\sum_{j}M_{j}T_{c2j}+\sum_{k}O_{k}T_{c3k}\right)$
${\left(P_{c}-1.3705\right)}^{-0.5}-0.10022=\sum_{i}N_{i}p_{c1i}+\sum_{j}M_{j}p_{c2j}$	${\left(P_{c}-5.9827\right)}^{-0.5}-0.108998\;\;=\sum_{i}N_{i}P_{c1i}+\sum_{j}M_{j}P_{c2j}+\sum_{k}O_{k}P_{c3k}$
$V_{c}+0.00435=\sum_{i}N_{i}v_{c1i}+\sum_{j}M_{j}v_{c2j}$	$V_{c}-7.95=\sum_{i}N_{i}V_{c1i}+\sum_{j}M_{j}V_{c2j}+\sum_{k}O_{k}V_{c3k}$
**Joback & Reid [63]**	**Marrero-Pardillo [65]**
$T_{b}=198.2+\sum n_{i}T_{bi}$	$T_{b}=204.66+\sum$
$T_{c}=T_{b}\;{\left[0.584+0.965\;\left(\sum n_{i}T_{ci}\right)-{\left(\sum n_{j}T_{cj}\right)}^{2}\right]}^{-1}$	$T_{c}=T_{b}/\left[0.5851-0.9286\sum -\sum^{2}\right]$
$P_{c}={\left(0.113+0.0032\;n_{A}-\sum n_{i}P_{ci}\right)}^{-2}$	$P_{c}={\left(0.1285-0.0059\;n_{A}-\sum \right)}^{-2}$
$V_{c}=17.5+\sum$	$V_{c}=25.1+\sum$

**Table 2:** The RK-Aspen EOS with the van der Waals mixing rules and RK-Aspen combining rules for two temperature-independent binary interaction parameters $k_{aij}=k_{aij}^{0}$ and $k_{bij}=k_{bij}^{0}$.

		Equation of State
RK-Aspen	$\;\;P=\frac{RT}{V-b}-\frac{a\left(T\right)}{V\left(V+b\right)}$	$a=0.42748\frac{R^{2}T_{c}^{2}}{P_{c}}\times \alpha \left(m_{i},\eta_{i},\;T_{ri}\right)$ $b=0.08664\frac{RT_{c}}{P_{c}}$
		$\alpha \left(m_{i},\eta_{i},\;T_{ri}\right)={\left[1+m_{i}\left(1-T_{ri}^{1/2}\right)-\eta_{i}\left(1-T_{ri}\right)(0.7-T_{ri})\right]}^{2}$
	Mixing Rules	
	$a=\sum \sum x_{i}x_{j}a_{ij}$	
van der Waals (RK-Aspen)	$b=\sum \sum x_{i}x_{j}b_{ij}$	
	$a_{ij}={\left(a_{ii}a_{jj}\right)}^{1/2}\left(1-k_{aij}\right)$	$k_{aij}=k_{aij}^{0}+k_{aij}^{1}\frac{T}{1000}$
	$b_{ij}=\frac{\left(b_{ii}b_{jj}\right)}{2}\left(1-k_{bij}\right)$	$k_{bij}=k_{bij}^{0}+k_{bij}^{1}\frac{T}{1000}$

**Table 3:** Experimental gaseous-liquid equilibrium data for the binary pair’s organic liquid products compounds-_i_-CO_2_ used to compute the binary interaction parameters of RK-Aspen EOS [74,75,76,77,78,79,80,81].

CO_2_^+^	N	T [K]	P [bar]	References
Decane	29	319.11–372.94	34.85–160.60	Jimenez-Gallegos et al. (2006)
Undecane	18	314.98–344.46	23.73–133.88	Camacho-Camacho et al. (2007)
Tetradecane	2	344.28	155.54–162.99	Gasem et al. (1989)
Pentadecane	22	293.15–353.15	5.60–139.40	Secuianu et al. (2010)
Hexadecane	12	314.14–333.13	80.65–148.70	D’Souza et al. (1988)
Octadecane	12	534.86–605.36	10.16–61.90	Kim et al. (1985)
Palmitic acid	10	423.20–473.20	10.10–50.70	Yau et al. (1992)
Oleic acid	16	313.15–353.15	101.70–300.20	Bharath et al. (1992)

**Table 4:** Experimental gaseous-liquid equilibrium data for the binary systems oleic acid-CO_2_, squalene-CO_2_, and triolein-CO_2_ used to compute the binary interaction parameters of RK-Aspen EOS [81,82,83,84,85].

CO_2_^+^	T [K]	P [bar]	N	References
**Triolein**	333.15, 353.15	200-500	8	Weber et al. (1999)
	313.15–333.15	153.4–310.0	8	Bharath et al. (1992)
**Oleic acid**	313–333	72.1–284.1	12	Zou et al. (1990)
	313.15–353.156	101.7–300.2	16	Bharath et al. (1992)
**Squalene**	313–333	100–350	11	Hernandez et al. (2010)
	333.15–363.15	100–350	12	Brunner et al. (2009)

**Table 5:** Selected methods to predict the normal boiling temperature (T_b_) of hydrocarbons classes (*n*-alkanes, alkenes, unsubstituted cyclics, substituted cyclics, and aromatics), of all the compounds present in OLP, obtained by thermal catalytic cracking of palm oil at 450 °C, 1.0 atmosphere, with 10% (wt.) Na_2_CO_3_ [17].

Class of Hydrocarbons	N	ARD [%]	S [%]	R [%]	Methods
*n*-Alkanes	27	−2.806	1.943	6.797	Marrero-Gani
Alkenes	19	−0.848	1.206	5.396	Marrero-Gani
Unsubstituted cyclics	9	−2.001	4.815	13.720	Constantinou-Gani
Substituted cyclics	62	−0.546	2.208	10.604	Constantinou-Gani
Aromatics	28	−0.214	1.916	7.021	Joback

**Table 6:** Selected methods to predict the critical temperature (T_c_) of hydrocarbons classes (*n*-alkanes, alkenes, unsubstituted cyclics, substituted cyclics, and aromatics), of all the compounds present in OLP, obtained by thermal catalytic cracking of palm oil at 450 °C, 1.0 atmosphere, with 10% (wt.) Na_2_CO_3_ [17].

Class of Hydrocarbons	N	ARD [%]	S [%]	R [%]	Methods
*n*-Alkanes	15	−0.685	0.157	0.496	Marrero-Gani
Alkenes	14	0.74	0.544	2.072	Marrero-Gani
Unsubstituted cyclics	7	−0.839	2.113	5.364	Marrero-Gani
Substituted cyclics	13	0.152	1.089	3.364	Marrero-Gani
Aromatics	31	−1.192	2.233	8.415	Constantinou-Gani

**Table 7:** Selected methods to predict the critical pressure (P_c_) of hydrocarbons classes (*n*-alkanes, alkenes, unsubstituted cyclics, substituted cyclics, and aromatics), of all the compounds present in OLP, obtained by thermal catalytic cracking of palm oil at 450 °C, 1.0 atmosphere, with 10% (wt.) Na_2_CO_3_ [17].

Class of Hydrocarbons	N	ARD [%]	S [%]	R [%]	Methods
*n*-alkanes	17	3.749	2.12	6.996	Marrero-Pardillo
alkenes	16	0.353	2.781	10.809	Marrero-Gani
Unsubstituted cyclics	7	−0.47	2.355	6.593	Joback
Substituted cyclics	14	1.537	2.939	12.782	Constantinou-Gani
Aromatics	18	0.461	2.035	9.486	Marrero-Gani

**Table 8:** Selected methods to predict the critical volume (V_c_) of hydrocarbons classes (*n*-alkanes, alkenes, unsubstituted cyclics, substituted cyclics, and aromatics), of all the compounds present in OLP, obtained by thermal catalytic cracking of palm oil at 450 °C, 1.0 atmosphere, with 10% (wt.) Na_2_CO_3_ [17].

Class of Hydrocarbons	N	ARD [%]	S [%]	R [%]	Methods
*n*-alkanes	8	−0.23	0.681	2.070	Marrero-Gani
alkenes	16	−0.113	1.210	4.013	Marrero-Pardillo
Unsubstituted cyclics	6	−1.024	1.359	2.215	Joback
Substituted cyclics	14	−0.318	4.832	12.717	Marrero-Gani
Aromatics	19	0.034	1.914	6.785	Constantinou-Gani

**Table 9:** Selected methods to predict the acentric factor (ω) of hydrocarbons classes (*n*-alkanes, alkenes, unsubstituted cyclics, substituted cyclics, and aromatics), of all the compounds present in OLP, obtained by thermal catalytic cracking of palm oil at 450 °C, 1.0 atmosphere, with 10% (wt.) Na_2_CO_3_ [17].

Class of Hydrocarbons	N	ARD [%]	S [%]	R [%]	Methods
*n*-alkanes	16	0.033	1.943	7.703	Han-Peng
alkenes	15	0.976	5.518	20.825	Han-Peng
Unsubstituted cyclics	7	2.822	2.555	7.374	Vetere
Substituted cyclics	16	1.843	3.539	15.089	Vetere
Aromatics	14	1.323	2.105	9.39	Vetere

**Table 10:** Estimated/Predicted values of thermo-physical (T_b_), critical properties (T_c_, P_c_, V_c_), and acentric factor (ω) of olive oil key (oleic acid, squalene, triolein) compounds [81,82,83,84,85].

Compounds	Cas Number	MW	T_b_ [°C]	T_C_ [°C]	P_C_ [kPa]	Vc [m^3^/kmol]	ω
**Triolein**	122-32-7	885.00	616.7	673.9	468.2	3.022	1.686
**Oleic acid**	122-80-1	282.2	353.7	579.4	1388.0	1.101	1.0787
**Squalene**	7683-64-9	410.7	401.2	564.9	653.0	2.052	1.398

**Table 11:** RK-Aspen EOS temperature-independent binary interaction parameters adjusted with experimental phase equilibrium data for the binary systems hydrocarbons-_i_-CO_2_ and carboxylic acids-_i_-CO_2_.

CO_2_^+^	T [K]	$\;k_{aij}=k_{aij}^{0}$	$\;k_{bij}=k_{bij}^{0}$	AADx	AADy
Undecane	314.98	0.116458	−0.008014	0.0029	0.0003
	344.46	0.103282	−0.029465	0.0030	0.0036
Tetradecane	344.28	0.099874	−0.000546	0.0003	0.0011
Pentadecane	313.15	0.093344	0.026454	0.0125	0.0020
	333.15	0.101805	0.014904	0.0067	0.0030
Hexadecane	314.14	0.083111	−0.075317	0.0117	0.0090
	333.13	0.082146	−0.081056	0.0013	0.0034
Octadecane	534.86	0.246616	0.073306	0.0010	0.0024
	605.36	0.107125	0.015525	0.0002	0.0064
Palmitic acid	423.20	−0.179556	−0.042625	0.0037	8.93 × 10^−5^
	473.20	−0.059218	−0.013329	0.0008	0.0002
Oleic acid	313.15	0.110902	0.132527	0.0039	0.0031
	333.15	0.116604	0.054485	0.0039	0.0035
	353.15	0.117892	0.049413	0.0049	0.0046

**Table 12:** RK-Aspen EOS $k_{aij}=k_{aij}^{0}\;$ and $\;k_{bj}=k_{bij}^{0}$ adjusted with experimental phase equilibrium data for the binary systems of olive oil key compounds-_i_-CO_2_.

CO_2_^+^	T (K)	$\;k_{aij}=k_{aij}^{0}$	$\;k_{bij}=k_{bij}^{0}$	AADx	AADy
**Triolein**	313.15 *^(a)^*	0.071209	0.099779	0.0094	0.0027
	333.15 *^(a)^*	0.077653	0.096221	0.0126	0.0030
	333.15 *^(b^*^)^	0.078059	0.083746	0.0088	0.0003
	353.15 *^(b)^*	0.103763	0.132745	0.0050	0.0001
**Squalene**	333.15 *^(c)^*	0.054090	−0.023325	0.0022	0.0025
	363.15 *^(c)^*	0.047825	−0.032640	0.0031	0.0014
	313 *^(e)^*	0.065395	−0.030832	0.0151	0.0016
	333 *^(e)^*	0.067249	−0.032589	0.0128	0.0013
**Oleic acid**	313.15 *^(a)^*	0.115801	0.130956	0.0199	0.0031
	333.15 *^(a)^*	0.116604	0.054485	0.0084	0.0035
	353.15 *^(a)^*	0.117892	0.049413	0.0062	0.0046
	313.15 *^(d)^*	0.070093	−0.006360	0.0094	0.0051
	333.15 *^(d)^*	0.089088	0.041100	0.0002	0.0067

*^(a)^*-Bharath et al. (1992), *^(b)^*-Weber et al. (1999), *^(c)^*-Brunner et al. (2009), *^(d)^*-Zou et al. (1990), *^(e)^*-Hernandez et al. (2010).

**Table 13:** RK-Aspen EOS temperature-independent binary interaction parameters adjusted with experimental high-pressure phase equilibrium data for the model systems oleic acid(1)-squalene(3)-triolein(2)-CO_2_(4).

$\;k_{ij}\;$	1–2	1–3	1–4	2–3	2–4	3–4	RMSDx	RMSDy
FFA in feed = 2.9 [wt.%], T[K] = 313								
$k_{aij}^{0}$	−1.44168	1.00000	−1.56772	−0.47399	0.08331	−0.54788	0.0014	2.0 × 10^−6^
$k_{bij}^{0}$	0.94017	−0.12023	−2.27950	1.00000	0.26809	−0.52108		
FFA in feed = 2.9 [wt.%], T[K] = 323								
$k_{aij}^{0}$	−1.78375	1.00000	−2.19466	−1.26916	0.06593	−0.90195	0.0017	2.8 × 10^−5^
$k_{bij}^{0}$	1.97388	0.32731	−3.32627	−0.36925	0.31781	−0.87789		
FFA in feed = 5.2 [wt.%], T[K] = 338								
$k_{aij}^{0}$	2.44416	−0.68874	2.65877	−0.07842	0.00445	0.40698	3.0E-07	2.0 × 10^−8^
$k_{bij}^{0}$	2.16063	0.99919	1.77156	0.08648	0.37099	−1.22870		
FFA in feed = 5.2 [wt.%], T[K] = 353								
$k_{aij}^{0}$	0.05504	0.13002	0.14571	0.16947	0.08930	0.43896	0.0058	7.4 × 10^−7^
$k_{bij}^{0}$	0.12297	−0.66975	0.00107	−0.91096	0.18358	0.66131		
FFA in feed = 7.6 [wt.%], T[K] = 313								
$k_{aij}^{0}$	−0.39597	0.95245	−0.41348	−0.40686	0.06993	−0.16959	0.0010	0.0002
$k_{aij}^{0}$	0.74176	−4.75749	−0.76430	0.70767	0.27402	0.22365		
FFA in feed = 15.3[wt.%], T[K] = 338								
$k_{aij}^{0}$	1.89568	0.99648	2.17462	0.31810	0.06235	−0.22787	0.0007	6.9 × 10^−5^
$k_{aij}^{0}$	0.75657	0.51874	2.50669	0.92458	0.20835	0.54779		

**Table 14:** State conditions (T, P) by high-pressure phase equilibrium data for the system olive oil-CO_2_ [90].

FFA [wt.%]	T [K]	P [bar]	N	Reference
2.9	313	138–275	4	Simões and Brunner (1996)
	323	182–257	3	
5.2	338	190–280	2	
	353	210–298	3	
7.6	313	180–281	3	
	323	179–212	2	
15.3	313	180–302	3	
	338	21–259	2	
	353	212–303	3	

**Table 15:** The average absolute deviation (AAD) between the predicted and experimental high-pressure phase equilibrium data for the model systems oleic acid(1)-squalene(3)-triolein(2)-CO_2_(4).

AAD									
FFA in Feed [wt.%]	T [K]	x_1_	x_2_	x_3_	x_4_	y_1_	y_2_	y_3_	y_4_
2.9	313	0.0062	0.1906	0.0017	0.1828	0.0000	0.0002	0.0000	0.0003
2.9	323	0.0164	0.2310	0.0028	0.2118	0.0020	0.0027	0.0006	0.0027
5.2	338	0.0000	0.0000	0.0000	0.0000	0.0000	0.0000	0.0000	0.0000
5.2	353	0.0215	0.7240	0.0033	0.7082	0.0001	0.0001	0.0000	0.0001
7.6	313	0.0164	0.1274	0.0014	0.1097	0.0093	0.0225	0.0009	0.0324
15.3	338	0.0090	0.0908	0.0003	0.0816	0.0053	0.0059	0.0000	0.0112

**Table 16:** Estimated RK-Aspen-EOS binary interaction parameters for multicomponent system FFA (oleic acid)(l)-Triglyceride (triolein)(2)-Squalene(3)-CO_2_(4).

$\;k_{ij}\;$	1–2	1–3	1–4	2–3	2–4	3–4	RMSDx	RMSDy
FFA in feed *=* 2.9 and 7.6 [wt.%], T [K] = 313								
$k_{aij}^{0}$	−0.150477	−0.297646	−0.111520	−0.602362	0.074580	−0.802333	0.0138	0.0009
$k_{bij}^{0}$	0.286959	−0.614753	−0.372917	0.992085	0.127803	−0.803741		

**Table 17:** The distribution coefficients-Ki of key compounds FFA (l), triglyceride (2), and squalene (3), expressed on a solvent free basis, by the experimental high-pressure phase equilibria for the multicomponent system olive oil-CO_2_.

FFA in Feed [wt.%]/T [K]	P [bar]	K_1_ × 10^2^		K_2_ × 10^2^		K_3_ × 10^2^	
		Exp	Est	Exp	Est	Exp	Est
2.9/313	138	1.09	1.08	0.09	0.09	1.71	1.71
	176	2.88	2.87	0.36	0.36	4.27	4.26
	208	4.01	4.01	0.52	0.52	4.75	4.73
	275	4.50	4.51	0.81	0.81	5.95	5.98
2.9/323	182	1.93	1.97	0.10	0.10	2.54	2.55
	206	2.68	2.83	0.25	0.25	4.03	4.30
	257	4.17	3.99	0.70	0.71	4.77	4.59
5.2/338	190	1.08	1.08	0.08	0.08	1.26	1.26
	280	4.59	4.59	0.88	0.88	5.34	5.34
5.2/353	210	2.89	2.87	0.18	0.18	2.11	2.11
	260	4.76	4.80	0.44	0.44	3.07	3.10
	298	7.11	7.08	0.88	0.86	5.13	5.17
7.6/313	180	2.32	2.48	0.28	0.28	3.45	3.67
	208	3.33	3.04	0.57	0.54	4.06	3.83
	281	4.64	4.73	0.90	0.96	5.02	4.95
15.3/338	215	2.65	2.71	0.54	0.55	7.60	7.61
	259	2.67	2.60	1.08	1.07	5.16	5.17

## Data Availability

Not applicable.

## Supplementary Materials

The following are available online. Table S1: Chemical composition of OLP. Table S2: Estimated/Predicted values of thermo-physical (T_b_), critical properties (T_c_, P_c_, V_c_), and acentric factor (ω) of chemical compounds present in OLP.
