# Supplementary material for: Simulation of Organic Liquid Products Deoxygenation by Multistage Countercurrent Absorber/Stripping Using CO2 as Solvent with Aspen-HYSYS: Thermodynamic Data Basis and EOS Modeling
Source: Molecules. 2021 Jul 20;26(14):4382. doi: 10.3390/molecules26144382 (PMC8307044; doi:10.3390/molecules26144382)
Supplement: Supplementary file 1 [file molecules-26-04382-s001.zip › molecules-1225853-supplementary.pdf]

molecules

Supplementary Materials

Simulation of Organic Liquid Products Deoxygenation by Multistage Countercurrent Absorber/Stripping Using CO_2 as Solvent with Aspen-HYSYS: Thermodynamic Data Basis and EOS Modeling

Elinéia Castro Costa [1], Welisson de Araújo Silva [1], Eduardo Gama Ortiz Menezes [2], Marcilene Paiva da Silva [2], Vânia Maria Borges Cunha [2], Andréia de Andrade Mâncio [1], Marcelo Costa Santos [1], Sílvio Alex Pereira da Mota [1], Marilena Emmi Araújo [2] and Nélio Teixeira Machado [1,2,3,*]

[1] Graduate Program of Natural Resources Engineering of Amazon, Rua Corrêa N° 1, Campus Profissional-UFPA, Belém 66075-110, Pará, Brazil; elineia.costa.ec@gmail.com (E.C.C.); wasilva89@hotmail.com (W.d.A.S.); dedeiaam@yahoo.com.br (A.d.A.M.); marceloenqui@bol.com.br (M.C.S.); silviomota@unifesspa.edu.br (S.A.P.d.M.)
[2] Graduate Program of Chemical Engineering, Rua Corrêa N° 1, Campus Profissional-UFPA, Belém 66075-110, Pará, Brazil; ortizegom@hotmail.com (E.G.O.M.); arci_paiva@hotmail.com (M.P.d.S.); vaniacunha21@hotmail.com (V.M.B.C.); meaaraujo@gmail.com (M.E.A.)
[3] Faculty of Sanitary and Environmental Engineering, Rua Corrêa N° 1, Campus Profissional-UFPA, Belém 66075-123, Pará, Brazil
* Correspondence: proderna@ufpa.br or ppgeq@ufpa.br; Tel.: +55-91-984620325

Table S1. Chemical composition of OLP, obtained by thermal catalytic cracking of palm oil at 450 °C, 1.0 atmosphere, with 10% (wt.) Na_2CO_3 [17], used to predict the thermo-physical (T_b), critical properties (T_c, P_c, V_c), and acentric factor ( ) of all the compounds present in OLP.

Chemical Compounds	Molecular Formula	CAS Number
Metil-Cyclooctane	C_9H_{18}	1502-38-1
1-Butyl-1-Cyclohexene	$C_{10}H_{18}$	3282-53-9
3-Isobutyl-1-Cyclohexene	$C_{10}H_{18}$	4104-56-7
1-Decene	$C_{10}H_{20}$	872-5-9
Decane	$C_{10}H_{22}$	124-18-5
1-Hexil-Cyclopentene	$C_{11}H_{20}$	Not Available
1-Undecene	$C_{11}H_{22}$	821-95-4
(E)-2-Undecene	$C_{11}H_{22}$	693-61-8
(Z)-2-Undecene	$C_{11}H_{22}$	Not Available
Undecane	$C_{11}H_{24}$	1120-21-4
1-Dodecene	$C_{12}H_{24}$	112-41-4
1-Tridecene	$C_{13}H_{26}$	2437-56-1
Cyclotridecane	$C_{13}H_{26}$	295-02-3
Tridecane	$C_{13}H_{28}$	629-50-5
1-Octyl-Cyclohexene	$C_{14}H_{26}$	15232-87-8
1-Tetradecene	$C_{14}H_{28}$	1120-36-1
Cyclotetradecane	$C_{14}H_{28}$	295-17-0
Tetradecane	$C_{14}H_{30}$	629-59-4
1-Nonyl-1-Cyclohexene	$C_{15}H_{28}$	15232-88-9
1-Pentadecene	$C_{15}H_{30}$	13360-61-7
Nonylcyclohexane	$C_{15}H_{30}$	2883-02-5
Cyclopentadecane	$C_{15}H_{30}$	295-48-7
Pentadecane	$C_{15}H_{32}$	629-62-9

(Z)-7-Hexadecene	$C_{16}H_{32}$	Not Available
(Z)-3-Hexadecene	$C_{16}H_{32}$	34303-81-6
1-Hexadecene	$C_{16}H_{32}$	629-73-2
Cyclohexadecane	$C_{16}H_{32}$	295-65-8
Hexadecane	$C_{16}H_{34}$	544-76-3
1-Heptadecene	$C_{17}H_{34}$	6765-39-5
(E)-9-Octadecene	$C_{18}H_{36}$	5557-31-3
Octadecane	$C_{18}H_{38}$	593-45-3
(E)-9-Eicosene	$C_{20}H_{40}$	Not Available
(Z)-9-Tricosene	$C_{23}H_{46}$	27519-2-4
Caproic Acid	$C_6H_{12}O_2$	142-62-1
Glycerol	$C_3H_8O_3$	56-81-5
N-Nonanoic Acid	$C_9H_{18}O_2$	112-05-0
Capric Acid	$C_{10}H_{20}O_2$	334-48-5
Lauric Acid	$C_{12}H_{24}O_2$	143-07-7
Myristc Acid	$C_{14}H_{28}O_2$	544-63-8
2-Nonadecanone	$C_{19}H_{38}O$	629-66-3
Ethyl Palmitate	$C_{18}H_{36}O_2$	628-97-7
Octadecan-4-one	$C_{18}H_{36}O$	Not Available
Palmitic Acid	$C_{16}H_{32}O_2$	57-10-3
Z-10-Octadecene-1-ol Acetate	$C_{20}H_{38}O_2$	Not Available
1-Eicosanol	$C_{20}H_{42}O$	629-96-9
Ethyl Oleate	$C_{20}H_{38}O_2$	111-62-6
Linoleic Acid	$C_{18}H_{32}O_2$	60-33-3
Oleic Acid	$C_{18}H_{36}O$	112-80-1
Stearic Acid	$C_{18}H_{36}O_2$	57-11-4
8-Octadecanone	$C_{18}H_{36}O$	79246-41-6

Table S2. Estimated/Predicted values of thermo-physical (T_b), critical properties (T_c, P_c, V_c), and acentric factor ( ) of chemical compounds present in OLP obtained by thermal-catalytic cracking of palm oil, as described by Mâncio et al. [17].

**Chemical Compounds**	**T_b [K]**	**T_c [K]**	**P_c [bar]**	**V_c [cm³/mol]**	**ω**
Metil-Cyclooctane	446.111 [b]	639.678 [c]	24.958 [b]	468.72 [c]	0.3794 [h]
1-Butyl-1-Cyclohexene	457.679 [b]	593.736 [c]	24.124 [b]	515.94 [c]	1.0313 [h]
3-Isobutyl-1-Cyclohexene	439.701 [b]	588.010 [c]	23.996 [b]	518.05 [c]	0.7739 [h]
1-Decene	440.000 [a]	615.724 [c]	22.200 [a]	584.00 [a]	0.4869 [f]
Decane	447.200 [a]	617.70 [a]	21.100 [a]	594.89 [c]	0.4920 [a]
1-Hexil-Cyclopentene	476.755 [b]	583.790 [c]	22.342 [b]	522.98 [c]	1.6624 [h]
1-Undecene	466.000 [a]	636.280 [c]	20.073 [d]	638.00 [d]	0.5313 [f]
(E)-2-Undecene	469.338 [c]	639.873 [c]	16.116 [d]	689.90 [d]	0.5356 [f]
(Z)-2-Undecene	469.338 [c]	639.873 [c]	16.116 [d]	689.90 [d]	0.5356 [f]
Undecane	469.080 [a]	639.00 [a]	19.500 [a]	651.17 [c]	0.5300 [a]
1-Dodecene	486.200 [a]	655.158 [c]	19.300 [a]	694.60 [d]	0.5747 [f]
1-Tridecene	505.000 [a]	672.609 [c]	17.047 [d]	751.20 [d]	0.6171 [f]
Cyclotridecane	507.685 [b]	739.275 [c]	23.726 [e]	641.50 [e]	0.2895 [h]
Tridecane	508.620 [a]	675.00 [a]	16.800 [a]	763.73 [c]	0.6170 [a]
1-Octyl-Cyclohexene	494.340 [b]	673.018 [c]	20.150 [b]	741.06 [c]	0.5579 [h]
1-Tetradecene	524.200 [a]	688.836 [c]	15.785 [d]	807.80 [d]	0.6585 [f]
Cyclotetradecane	522.830 [b]	756.412 [c]	17.625 [b]	689.50 [e]	0.1939 [h]
Tetradecane	523.000 [a]	693.00 [a]	16.470 [c]	830.00 [a]	0.6430 [a]

1-Nonyl-1-Cyclohexene	539.343 [b]	689.216 [c]	15.891 [b]	797.34 [c]	0.8912 [h]
1-Pentadecene	541.540 [a]	703.998 [c]	14.657 [d]	864.40 [d]	0.6990 [f]
Nonylcyclohexane	545.000 [a]	728.078 [c]	16.056 [b]	814.74 [c]	0.5544 [h]
Cyclopentadecane	536.929 [b]	772.366 [c]	20.755 [e]	737.50 [e]	0.2864 [h]
Pentadecane	540.000 [a]	708.00 [a]	14.800 [a]	876.29 [c]	0.6860 [a]
(Z)-7-Hexadecene	560.902 [c]	734.435 [c]	15.046 [d]	1112.2 [d]	0.7426 [f]
(Z)-3-Hexadecene	560.902 [c]	734.435 [c]	15.046 [d]	1112.2 [d]	0.7426 [f]
1-Hexadecene	547.200 [a]	703.998 [c]	13.647 [d]	921.00 [d]	0.7387 [f]
Cyclohexadecane	550.118 [b]	787.290 [c]	19.475 [e]	785.50 [e]	0.2816 [h]
Hexadecane	554.000 [a]	723.00 [a]	14.000 [a]	932.57 [c]	0.7170 [a]
1-Heptadecene	559.440 [c]	731.631 [c]	12.737 [d]	977.60 [d]	0.7776 [f]
(E)-9-Octadecene	585.099 [c]	758.833 [c]	13.052 [d]	1225.4 [d]	0.8194 [f]
Octadecane	589.300 [a]	747.000 [a]	12.950 [a]	1045.13 [c]	0.8178 [f]
(E)-9-Eicosene	591.816 [b]	769.696 [c]	11.429 [d]	1338.6 [d]	0.8933 [f]
(Z)-9-Tricosene	620.891 [b]	800.665 [c]	9.508 [d]	1508.4 [d]	0.9993 [f]
Caproic Acid	477.000 [a]	660.200 [a]	33.800 [a]	413.00 [a]	0.7300 [a]
Glycerol	563.150 [a]	850.000 [a]	75.000 [a]	-	0.5130 [a]
N-Nonanoic Acid	527.150 [a]	710.700 [a]	25.140 [a]	562.89 [b]	0.7720 [a]
Capric Acid	530.000 [a]	722.100 [a]	22.500 [a]	618.65 [b]	0.8060 [a]
Lauric Acid	571.000 [a]	742.68 [b]	19.142 [b]	730,17 [b]	0.8689 [g]
Myristc Acid	588.311 [b]	762.508 [b]	16.356 [b]	841.69 [b]	0.9615 [g]
2-Nonadecanone	396.280 [e]	552.195 [e]	26.625 [e]	557.50 [e]	0.5575 [h]
Ethyl Palmitate	615.350 [a]	822.849 [e]	13.577 [b]	1065.50 [c]	0.8980 [g]
Octadecan-4-one	373.400 [e]	52.357 [e]	29.473 [e]	501.50 [e]	0.5698 [h]
Palmitic Acid	612.150 [a]	780.381 [b]	14.177 [b]	953.21 [b]	1.0281 [g]
Z-10-Octadecene-1-ol Acetate	723.65 [d]	896.254 [e]	12.6987 [c]	1158.27 [b]	0.9638 [h]
1-Eicosanol	742.70 [d]	808,000 [i]	11.500 [i]	1176.23 [c]	0.9100 [i]
Ethyl Oleate	717.050 [e]	888.080 [e]	12.699 [b]	1163.90 [c]	0.9009 [g]
Linoleic Acid	626.791 [b]	798.356 [b]	12.935 [b]	1032.51 [b]	0.9969 [g]
Oleic Acid	626.807 [b]	797.504 [b]	12.684 [b]	1048.62 [b]	1.0449 [g]
Stearic Acid	626.824 [b]	796.648 [b]	12.440 [b]	1064.73 [b]	1.0932 [g]
8-Octadecanone	372.700 [e]	525.993 [e]	30.933 [e]	492.50 [e]	0.5555 [h]

a-Yaws; b-Constantinou-Gani; c-Marrero-Gani; d-Marrero-Pardillo; e- Joback; f- Han-Peng; g-Ceriani; h-Vetere; i-Nikitin.
